# Isolation of single neuronal nuclei by phospho‐tau accumulation in Alzheimer’s disease brain

**DOI:** 10.1002/alz.093457

**Published:** 2025-01-03

**Authors:** Bowen Jin, Samuel Naik, Samantha L Kirkham, Xuyu Qian, Zinan Zhou, Derek Oakley, Matt P. Frosch, Bradley T. T Hyman, August Yue Huang, Michael B Miller

**Affiliations:** ^1^ Brigham and Women’s Hospital, Boston, MA USA; ^2^ Harvard Medical School, Boston, MA USA; ^3^ Boston Children’s Hospital, Boston, MA USA; ^4^ Boston children’s hospital, Boston, MA USA; ^5^ Massachusetts General Hospital, Boston, MA USA; ^6^ Neuropathology Service, C.S. Kubik Laboratory for Neuropathology, Massachusetts General Hospital, Harvard Medical School, Boston, MA USA; ^7^ MassGeneral Institute for Neurodegenerative Disease, Massachusetts General Hospital, Harvard Medical School, Charlestown, MA USA; ^8^ Massachusetts Alzheimer’s Disease Research Center, Charlestown, MA USA; ^9^ Massachusetts General Hospital, Harvard Medical School, Boston, MA USA; ^10^ MassGeneral Institute for Neurodegenerative Disease, Charlestown, MA USA

## Abstract

**Background:**

Alzheimer’s disease (AD) is a progressive neurodegenerative disorder characterized by the deposition of amyloid‐beta and hyperphosphorylated tau (P‐tau) proteins in the brain. P‐tau accumulates in neurons and is strongly associated with AD severity and affected brain regions. However, only a subset of neurons in AD exhibit tau pathology. The molecular mechanisms behind heterogeneous tau pathology and how it contributes to AD are not well understood.

**Method:**

We developed a fluorescence‐activated nuclear sorting (FANS) method to separate P‐tau+ neuronal nuclei from P‐tau‐ neuronal nuclei from the same brain tissue. To validate the specificity of P‐tau+ neurons, we mixed non‐AD control tissue with AD tissue and examined the origin of neuronal nuclei based on their genotype. We also subjected nuclei with P‐tau signal from FANS to immunofluorescence microscopy to examine the morphology and localization of P‐tau aggregates.

**Results:**

We observed disease‐specific P‐tau signal for nuclei in advanced AD (Braak stage V‐VI) cases. These P‐tau+ nuclei are highly distinguished from the P‐tau‐free nuclei. Confocal immunofluorescence microscopy showed P‐tau adherent to the outside of nuclei that exhibited P‐tau signal by FANS. We demonstrated that the nuclei sorting method based on P‐tau levels can highly enrich for P‐tau+ nuclei (>380 fold) and has a high accuracy of 98% based on the mixing experiments. In addition, we were able to obtain high‐quality single‐cell genome amplification for P‐tau‐sorted nuclei using primary template‐directed amplification (PTA), permitting single‐nucleus genome interrogation.

**Conclusion:**

We developed a highly efficient method using fluorescence‐activated nuclear sorting (FANS) to separate the population of P‐tau+ neuronal nuclei in AD brains. This method allows interrogation of human neuronal nuclei based on single‐cell tau pathology and enables single‐cell genomic studies of heterogeneous tau pathology in AD, toward insight into disease pathogenesis and therapeutic targets.

